# Supplementary material for: Opinions of physiotherapists at the University Teaching Hospital on assessing the psychological well-being of patients with stroke: A qualitative study
Source: PLOS Ment Health. 2025 Oct 29;2(10):e0000482. doi: 10.1371/journal.pmen.0000482 (PMC12798555; doi:10.1371/journal.pmen.0000482)
Supplement: Attachment — Submitted filename: Response to Reviewers.docx [file pmen.0000482.s001.docx]

Dear Editor

We are grateful for the opportunity to submit a revised draft of the manuscript “Opinions of Physiotherapists at the University Teaching Hospital on Assessing the Psychological Well-Being of Patients with Stroke: A Qualitative Study” to PLOS Mental Health. We would like to thank you and the reviewer for the time and effort you have put into providing your valuable feedback on our manuscript. We are very grateful to the reviewers for the insightful comments they have made on our paper. We have made changes to the manuscript as a result of the reviewer's suggestions. These changes have been highlighted in the manuscript by track changes.

Here is a point-by-point response to the reviewer’s comments and concerns.

**Comment 1:** The "Trustworthiness" section is particularly important, as it demonstrates the care taken to address some of the study's validity criteria. Although aspects related to the research team and reflexivity are described, it is necessary to present additional elements to better understand the position of the research team, as well as its relationship with the participants. This will enable a contextual understanding of the results and the proposed discussion. It is recommended that you review the COREQ guide criteria (Domain I) for qualitative studies, available on the EQUATOR network <https://www.equator-network.org/>

**Response:** Thank you for highlighting this. We have revised the section to include a description of the position of the research team and their relationship with the participants (Line 148-155).

**Comment 2:** Study participants: How were participants approached? How many people were invited but refused to participate or drop out? Where was the data collected? At the same hospital?

**Response:** Thank you for this guidance, information on how participants were approached (lines 100-102), and how many people were invited but refused to participate or drop out has been provided in the study (lines 109-111). Information on where the data was collected has been included (line 130-131).

**Comment 3:** Ethical Issues: Could you indicate which ethics committee approved the study protocol?

**Response:** The name of the ethics committee has been added (lines 175-176).

**Comment 4:** Results: If possible, and if it doesn't compromise the anonymity of the participants, it's probably very useful to describe the participants in a table, indicating the participant number (or ID). This number can be displayed at the end of each quote, making it easier to understand the participants' speech. Please check that in all textual quotes, the person (participant ID) speaking is indicated.

**Response:** We agree with you, the table describing participants has been included (lines 184-185). The participant ID has been attached to each quote.

**Comment 5:** Discussion: In the limitations section, the authors mentioned that the study involved ten physiotherapists from a single university teaching hospital. Could you expand on this point? How might it differ in other contexts? Perhaps, being a university hospital, they have a broader vision than others?

**Response:** The point in the limitation has been expanded to improve understanding (lines 332-336)
