# Supplementary material for: Opinions of physiotherapists at the University Teaching Hospital on assessing the psychological well-being of patients with stroke: A qualitative study
Source: PLOS Ment Health. 2025 Oct 29;2(10):e0000482. doi: 10.1371/journal.pmen.0000482 (PMC12798555; doi:10.1371/journal.pmen.0000482)
Supplement: Attachment — Submitted filename: Point by point response to reviewers comments.docx [file pmen.0000482.s002.docx]

Dear Editor

We would like to thank you and the reviewers for the time and effort you have put into providing your valuable feedback on our manuscript. We are very grateful to the reviewers for the insightful comments they have made on our paper. We have made changes to the manuscript as a result of the reviewer's suggestions. These changes have been highlighted in the manuscript by track changes.

Here is a point-by-point response to the reviewer’s comments and concerns.

**Comments to Reviewer 1**

**Comment 1:** There's just one detail: it would be important to standardize all citations, including the ID or participant number (in all citations) in a consistent and organized manner. Perhaps the ID number at the end of the citation would suffice, for example: "....XXX..." (ID 3).

**Response:** Thank you for the guidance. The citations have been revised as suggested.

**Comments to Reviewer 2**

**Comment 1:** Materials and methods: Nicely done overall, good level of detail. Suggested edit: page 6, lines 123-124. The term "rare and terrifying illness of the mind" sounds bizarre to me. If this is a quote from your reference, please use quotation marks. If this is the authors' phrasing, I suggest rewording to something like "...that lie on continuous dimensions and are not necessarily diagnosed mental illnesses."

**Response:** Thank you for this guidance. The sentence has been revised as suggested. It now reads, “Psychological problems were defined as problematic ways of thinking, feeling, and behaving that lie on continuous dimensions and are not necessarily diagnosed mental illnesses.” (page 6: 122-123)

**Comment 2**: Results: Table 1 compromises the anonymity of participants. For example, if someone in the physiotherapy department of your institution reads your manuscript, would they not be able to identify who is the 51-year-old male with 23 years of experience? I would recommend removing this table and providing this data in aggregated form (which you do in the first paragraph), rather than specifying the details of every participant individually.

**Response:** We agree with the concern raised. To address this, the table showing individual details has been removed.

**Comment 3:** Discussion & Conclusion: I recommend that authors reflect more deeply on their discussion point regarding the appropriate scope of physiotherapy (e.g., page 14, lines 289-291 and 296-298; page 16, lines 346-347). Imagine the opposite scenario: psychologists who are assessing patients' mental health should of course consider the impact of physical impairments (e.g., hemiparesis), but is it ethical for them to conduct physiotherapy assessments?

**Response:** The sentences have been changed to stress the importance of using screening assessments to identify patients who should be referred to psychologists for thorough assessment, diagnosis and care (page 14: 295-296, 298-304; page 16: 348-354).

**Comments 4:** In the discussion, it would be helpful to clarify what the authors recommend the extent of this psychological assessment would be. In many countries, psychological assessment is the responsibility of a psychologist with rigorous training in mental health, so this would be beyond the scope of what a physiotherapist would be trained to do. In my opinion, the results point towards a discussion about the benefits of multi-disciplinary care and the usefulness of physiotherapists being trained in identifying the need for a referral to psychology, which the authors have touched upon.

**Response:** Thank you for this guidance. The extent of psychological assessment has been clarified using the International Organization of Physical Therapy in Mental Health (IOPTMH) guidance, which stipulates that all physiotherapists should be able to identify psychological problems by performing assessment utilising psychological screening tools, clinical observation, and patient interviews, but not diagnose or implement treatment strategies. (page 13: line 283-286)

**Comments 5:** Page 16, line 341: confusing phrasing, some words appear to be mixed up.

**Response:** The sentence was revised and changed. It now reads, “Key factors contributing to inconsistencies in the assessment include lack of standard guidelines, lack of confidence, lack of competence and inadequate training. Considering that physiotherapists work with patients with stroke who exhibit psychological problems, they could benefit from training in identifying such problems to facilitate referrals to specialised clinicians for diagnosis and management.” (page 16; 345-347).
